# Tart Cherry Increases Lifespan in *Caenorhabditis elegans* by Altering Metabolic Signaling Pathways

**DOI:** 10.3390/nu12051482

**Published:** 2020-05-20

**Authors:** Shasika Jayarathne, Latha Ramalingam, Hunter Edwards, Siva A. Vanapalli, Naima Moustaid-Moussa

**Affiliations:** 1 Department of Nutritional Sciences, Obesity Research Institute, Texas Tech University, Lubbock, TX 79409, USA; shasika-har.udahawatte@ttu.edu (S.J.); latha.ramalingam@ttu.edu (L.R.); 2 Department of Biological Sciences, Texas Tech University, Lubbock, TX 79409, USA; hunter.edwards@ttu.edu (H.E.); siva.vanapalli@ttu.edu (S.A.V.); 3 Department of Chemical Engineering, Texas Tech University, Lubbock, TX 79409, USA

**Keywords:** *C. elegans*, tart cherry, lifespan, aging, oxidative stress, *daf-16*

## Abstract

Aging and healthspan are determined by both environmental and genetic factors. The insulin/insulin-like growth factor-1(IGF-1) pathway is a key mediator of aging in *Caenorhabditis elegans* and mammals. Specifically, DAF-2 signaling, an ortholog of human IGF, controls DAF-16/FOXO transcription factor, a master regulator of metabolism and longevity. Moreover, mitochondrial dysfunction and oxidative stress are both linked to aging. We propose that daily supplementation of tart cherry extract (TCE), rich in anthocyanins with antioxidant properties may exert dual benefits for mitochondrial function and oxidative stress, resulting in beneficial effects on aging in *C. elegans*. We found that TCE supplementation at 6 μg or 12 μg/mL, increased (*p* < 0.05) the mean lifespan of wild type N2 worms, respectively, when compared to untreated control worms. Consistent with these findings, TCE upregulated (*p* < 0.05) expression of longevity-related genes such as *daf-16* and *aak-2* (but not *daf-2* or *akt-1* genes) and genes related to oxidative stress such as *sod-2*. Further, we showed that TCE supplementation increased spare respiration in N2 worms. However, TCE did not change the mean lifespan of *daf-16* and *aak-2* mutant worms. In conclusion, our findings indicate that TCE confers healthspan benefits in *C. elegans* through enhanced mitochondrial function and reduced oxidative stress, mainly via the DAF-16 pathway.

## 1. Introduction

Aging has a significant influence on economy, health, and demography with healthy aging being a global challenge [1]. In 2018, ~16% of the United States population were 65 years old or over and expected to reach 23% by 2060 [2]. Globally, 9% of the population was old (over age 65) in 2019, which is expected to increase to 16% by 2050 [3]. Genetic and lifestyle factors contribute to aging and healthy lifespan. The effects of aging could be altered, and progression of aging could be slowed down [4]. 

Various factors contribute to aging such as defects in development and genetics, environmental factors, diseases, and reactive oxygen species (ROS), which cause harmful changes in cells and tissues [5]. Excessive ROS production causes oxidative stress, which is a predominant factor in the pathophysiology of various diseases and aging in human and other models [6] such as *Caenorhabditis elegans* (*C. elegans*) [7] and *Drosophila* [8]. Calorie restriction is the most established method for successfully extending lifespan of animals and model organisms such as *C. elegans* [9,10,11]. However, human studies are scarce [12], and the safety and effectiveness of calorie restriction for lifespan extension in humans is controversial [1]. Therefore, it is critical to identify dietary bioactive compounds that reduce ROS levels and thereby delay aging.

Among the pathways and genes involved in lifespan regulation, DAF-16 is homologous to mammalian forkhead box O1 (FOXO) transcription factor, and a key lifespan extension transcription factor that is highly conserved across a variety of organisms such as flies, worms, rodents, and humans [12,13]. *C. elegans* DAF-2 signaling, which is orthologous to insulin and insulin like growth factor-1 (IGF-1) in mammals, is also involved in metabolism and longevity but is negatively regulated by DAF-16/FOXO signaling [14]. Additionally, activation of AMP-activated protein kinase (AMPK), a conserved energy sensor in cells, extends lifespan of *C. elegans* [15]. In addition to these major pathways and genes, ROS is also involved in regulating longevity [16]. High ROS activity has been shown to be a major lifespan limiting factor in humans, *C. elegans*, and *Drosophila* [7,17,18,19]. Finally, in addition to the factors discussed above, mitochondria regulate different signaling and metabolic pathways, including ROS production and therefore plays a vital role in aging progression [20]. 

Dietary intervention is one of the effective non-genetic or pharmacological means to combat aging [21]. Botanicals like cranberry, strawberry, blueberry, sweet cherry, tart cherry, and curcumin all contain biologically active polyphenols, which may protect against diseases and promote health in animals, humans, and model organisms [22,23,24]. Moreover, cranberry [8,13,25], mulberry [26], and blueberry anthocyanins increase lifespan of *C. elegans* and *Drosophila* via different pathways [17,27,28]. Out of these, Montmorency tart cherry (*Prunus cerasus*) contains high concentration of anthocyanin flavonoids, and its consumption is known to provide numerous health benefits by reducing inflammation and production of ROS [29,30] as well as protecting against diabetes [31], cardiovascular diseases [32], muscle pain [33], and cancer [34]. 

Tart cherry (TC) possess increased antioxidant activity both in vivo and in vitro [35]. However, the mechanisms by which TC reverses effects of aging through antioxidant activities have not been studied yet and remain to be further elucidated. To investigate the effects of TC extract (TCE) on aging and healthspan, we used the nematode *C. elegans*, which is an excellent model for aging research [36] because of its short lifespan (2–3 weeks), experimental flexibility, and rapid turnover [37]. Furthermore, *C. elegans* behavior and physiological activities slow down with age similar to that of higher mammals, including humans [27]. Additionally, 83% of *C. elegans* proteome shows homology to the human proteome, and both humans and nematodes share significant age-related characteristics that can be used to study effects on health and aging [21,38]. 

In this paper, we determined mechanisms by which TCE extends lifespan in *C. elegans* using microfluidic devices for whole-life culture studies that allow efficient drug delivery compared to animals reared on agar-plates [39,40,41]. We demonstrated that TCE extends lifespan and healthspan of *C. elegans* by influencing the major DAF-16/FOXO lifespan regulating pathway. Consistent with this finding, TCE regulated upstream and downstream genes related to DAF-16 pathway, reduced the activity of genes related to ROS, and increased oxygen consumption rate. Therefore, our findings reveal protective anti-aging effects of TCE in vivo with robust translational potential in humans due to the feasibility of incorporating TCE into a typical human diet. 

## 2. Materials and Methods 

### 2.1. Preparation of Tart Cherry (TC) Extract

Frozen TC (Cherry Marketing Institute, Dewitt, MI, USA) was ground using motor and pestle to obtain TC extract (TCE). Extracted cherry juice was filtered using 0.22 µm filter and stored in −80 °C until further use. Amounts of TCE used are based on anthocyanin concentration. We used TCE with doses between 0–12 µg anthocyanin per mL media. TCE extract from frozen TC contains 533 ± 47 µg/g total anthocyanin [29], and 1 µL TCE contains 3 µg of anthocyanins as measured by high performance liquid chromatography mass-spectrometry (HPLC-MS) [35,42,43]. Additionally, TCE contains other phenolics, melatonin, vitamins A/C/E/K, niacin, beta carotene, lutein + zeaxanthin, and pantothenic acid [29,44]. To prevent oxidation, thermal degradation, chemical and biochemical changes, extracted TC was aliquoted (20 µL) and stored in −80 °C [45]. Aliquots of TCE were thawed only once and not refrozen, and TCE was replaced every day to prevent oxidation [45].

### 2.2. Strains and Growth Conditions

Worms (wild type N2, *daf-16*, *aak-2*) (Vanapalli lab, TTU, Lubbock, TX, USA) were maintained on solid nematode growth medium for two successive generations, as previously described [46]. On the third day, age-synchronized young adults were used for analyses. 

### 2.3. Lifespan Assay

At day 3, individual worms were suspended in liquid buffer (Nematode Growth Media (NGM)) and loaded into the micropillar chambers of a NemaLife microfluidic device using a syringe [40,41]. Each microfluidic device contained 30 individual chambers for animals to crawl, and one worm was loaded per chamber. Chambers were washed everyday with NGM to avoid progenies. Adult worms were fed with concentrated bacteria (100 mg/mL OP50) mixed with 3, 6, and 12 μg/mL TCE in the final mixture every day starting at day three until they all died. Chambers were rinsed everyday using liquid NGM media to wash out worm progenies and food was replenished. Worms were scored for survival every day. Worms killed during manipulation, wash errors, and bagging of worms exposed to contamination events were subsequently censored from analyses. At least three independent trials were performed for all conditions. Lifespan analyses were carried out using Kaplan–Meier statistical function using GraphPad Prism Version 7.04 software, with statistical significance considered at *p* < 0.05 using the log-rank (Mantel–Cox) test.

### 2.4. Gene Expression Analyses

Age synchronized worms ~500 µL was added into 60 mm plates. We estimated that each plate had approximately same number of worms ~1000 for RNA isolation. These N2 worms were grown in 3 mL of liquid axenic media for 7 days and then treated with or without tart cherry (6 µg/mL media, 12 µg/mL media) for 3 days, after which RNA was isolated using RNA isolation kit (Zymo Research Corp, Irvine, CA, USA) following manufacturers protocol. Same amount of total RNA was transcribed into cDNA using Maxima Reverse Transcription kit (Thermo Fisher, Waltham, CA, USA). Maxima powerup SYBR^TM^ Green Master Mix (Thermo Fisher, Waltham, CA, USA) was used for making cDNA to test targeted genes including *daf-16*, *daf-2*, *daf-18*, *aak-2*, *akt-1*, *lin-14*, *skn-1*, *ucp-4*, *sod-2/3* with *18s* housekeeping gene (Sigma-Aldrich, St. Louis, MO, USA).

### 2.5. Mitochondrial Respiration Analysis

To investigate TC-mediated changes in mitochondrial function, oxygen consumption rate (OCR) measurements were performed using the Seahorse XFe24 analyzer (Agilent, Santa Clara, CA, USA). Three days treated adult wild-type worms were washed twice in M9 buffer [47] and transferred into M9-filled wells (20 worms/well) in replicates of 4 per condition (i.e., 4 wells per strain). The experiment was repeated three times (*n* = 3) using the same conditions. To generate stable OCR measurements, 5 measurement cycles were performed for basal OCR, 9 cycles for maximal OCR following the addition of FCCP (10 μM final well concentration), and 5 cycles for non-mitochondrial OCR following the addition of sodium azide (40 mM final well concentration). OCR measurements were normalized to the number of worms per well. To avoid unstable OCR measurements, the final 3, 7, and 2 measurement cycles were used for the statistical analysis of basal, maximal, and non-mitochondrial OCR, respectively. 

### 2.6. Statistical Analysis

Differences in gene expression and OCR were detected with a one-way ANOVA with Tukey’s multiple comparison test using GraphPad Prism 7.04, with statistical significance considered at *p* < 0.05.

## 3. Results

### 3.1. Tart Cherry Extends Mean Lifespan of Wild Type N2 C. elegans in a Dose-Dependent Manner

**Figure 1:**
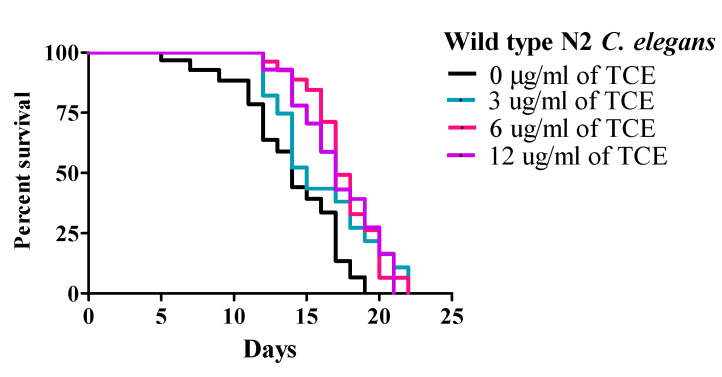
Tart cherry extract (TCE) extends lifespan in *Caenorhabditis elegans*. Treatment with 6 µg/mL (pink) and 12 µg/mL (purple) TCE increased mean lifespan (MLS) in wild type (N2) *C. elegans* (*p* < 0.05) by 16.83 ± 0.77 and 16.73 ± 0.67 days, respectively compared to control or no treatment (0 µg/mL, MLS 13.31 ± 0.61 days, black) or 3 µg/mL TCE (blue, MLS: 14.92 ± 0.58 days) grown at 20 °C. Each lifespan experiment was repeated at least three independent times with similar results.

Under these conditions, wild type (WT) worms had a mean lifespan (MLS) of 13.31 ± 0.61 days. The MLS of worms treated with TCE at concentrations of 3, 6, and 12 μg/mL were 14.92 ± 0.58, 16.83 ± 0.77, and 16.73 ± 0.67 days, respectively. While there was no difference with 3 μg/mL, both 6 and 12 μg/mL TCE significantly (*p* < 0.01) increased lifespan by 26.44% and 25.69%, respectively (Figure 1 and Supplementary Table S1), compared to no TCE treatment.

### 3.2. Tart Cherry Effects on Genes Related to Aging

**Figure 2:**
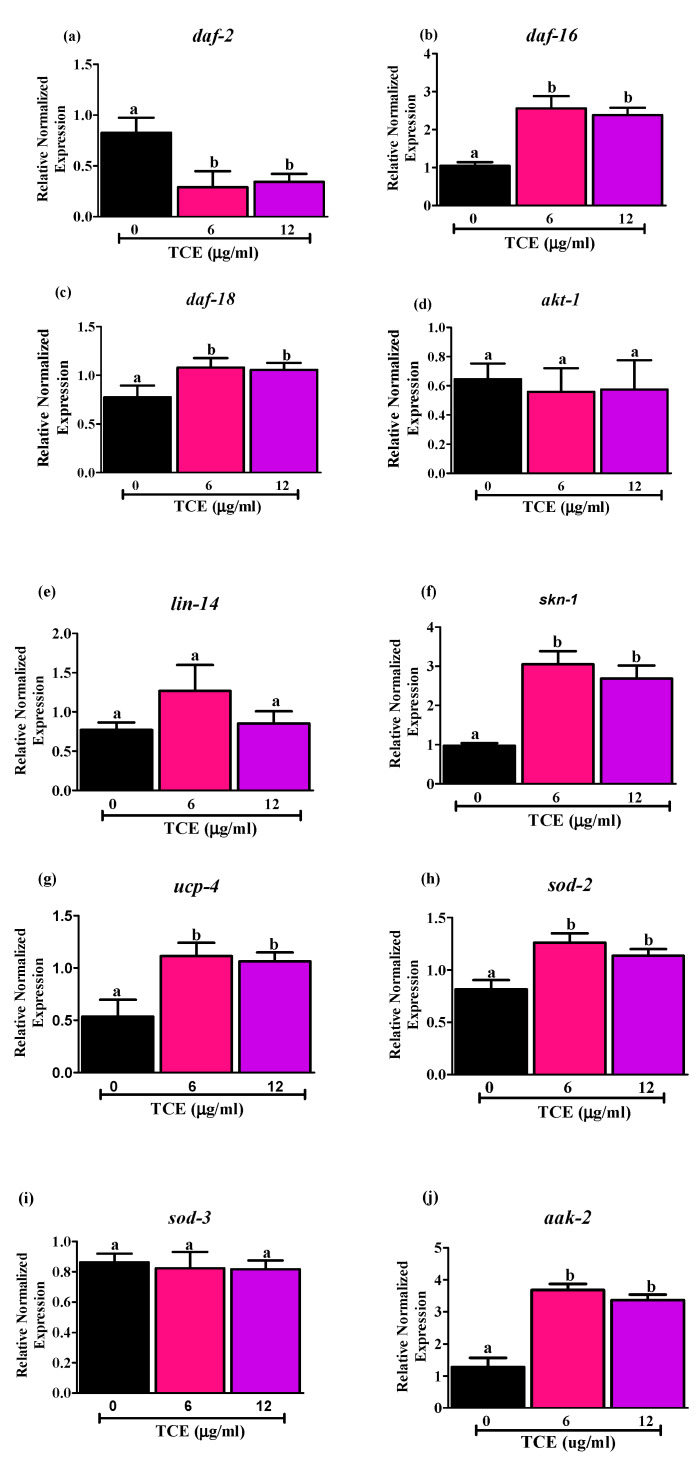
Effects of tart cherry extracts (TCE) on mRNA levels of (**a**) *daf-2*, (**b**) *daf-16*, (**c**) *daf-18*, (**d**) *akt-1*, (**e**) *lin-14*, (**f**) *skn-1*, (**g**) *ucp-4*, (**h**) *sod-2*, (**i**) *sod-3*, and (**j**) *aak-2*. mRNA expression of *daf-2*, *daf-16*, *daf-18*, *skn-1*, *ucp-4*, *sod-2*, *aak-2* was significantly different with 6 and 12 μg/mL TCE worms compared to not treated (0 μg/mL) worms (different letters (a, b) are significantly different, *p* < 0.05, One-way ANOVA, *n* = 5, five independent experiments with triplicates). There is no significant difference in the mRNA levels of *akt*, *lin-14* and *sod-3* in the 6 and 12 μg/mL treated worms compared to not treated worms (0 μg/mL). Synchronized worms (approx. *n* ~ 1000) grown in the axenic media were treated with 0, 6, and 12 μg/mL TCE, and gene expression analysis was performed at day 10 after 3 days of treatment.

To identify molecular mechanism(s) involved in promoting lifespan by TCE, we selected 6 and 12 μg/mL TCE that extended mean lifespan of WT worms in data discussed above. Using qRT-PCR, we evaluated the mRNA levels of *daf-2*, *age-1* ortholog of human phosphatidylinositol-4,5-bisphosphate 3-kinase, and *daf-16* genes in WT worms treated with 6 and 12 µg/mL TCE. Both 6 and 12 µg/mL TCE decreased mRNA expression of *daf-2* (Figure 2a) and increased mRNA expression of *daf-16* (Figure 2b). However, *age-1* gene, downstream target of insulin/insulin like growth factor signaling pathway (IIS), had very low expression (data not shown due to low expression levels).

Moreover, we measured the mRNA levels of *daf-18* gene (Figure 2c), a human phosphatase and tensin homolog (PTEN) tumor suppressor gene ortholog, which downregulates DAF-2 receptor pathway by inhibiting the activity of AGE-1 [48]. Here, we observed that *daf-18* was upregulated significantly by 6 and 12 μg/mL TCE, which may also be involved in down regulating IIS signaling and promoting DAF-16 nuclear localization [49]. Additionally, we measured two upstream DAF-16 inhibitors, serine-threonine protein kinase (*akt-1)* (Figure 2d) and *lin-14* (Figure 2e) and did not find any changes in expression levels of these genes following TCE treatment. This suggests that upregulation of upstream *daf-18* gene may inhibit the expression of *akt-1* and *lin-14*.

As antioxidant status and mitochondrial function are also important in aging, we measured the mRNA levels of markers involved in regulating mitochondrial homeostasis. Specifically, we measured levels of (i) SKiNhead (SKN-1), homolog to mammalian nuclear factor erythroid 2-related factor 2 (NRF-2); (ii) uncoupling protein-4 (UCP-4), mitochondrial uncoupling protein; (iii) SOD-2/3, a superoxide dismutase controlling ROS production; and (iv) aak-2, an AMP-activated protein kinase homolog known to directly regulate *daf-16* activity. Data in Figure 2 illustrate changes in expression of these genes following TCE treatment as shown by mRNA levels *of skn-1* (Figure 2f), *ucp-4* (Figure 2g), *sod-2* (Figure 2h), and *aak-2* (Figure 2j), all of which were significantly higher (*p* < 0.05) in the worms treated with 6 and 12 μg/mL TCE, compared to worms with no TCE treatments. By contrast, no differences were observed in mRNA levels for *sod-3* (Figure 2i) with TCE treatment at either dose tested. Collectively, our results indicate that TCE acts through the insulin/insulin-like signaling (IIS) pathways by directly or indirectly influencing the activities of the DAF-16 transcription factor. 

### 3.3. Effects of TCE on C. elegans Respiration

**Figure 3:**
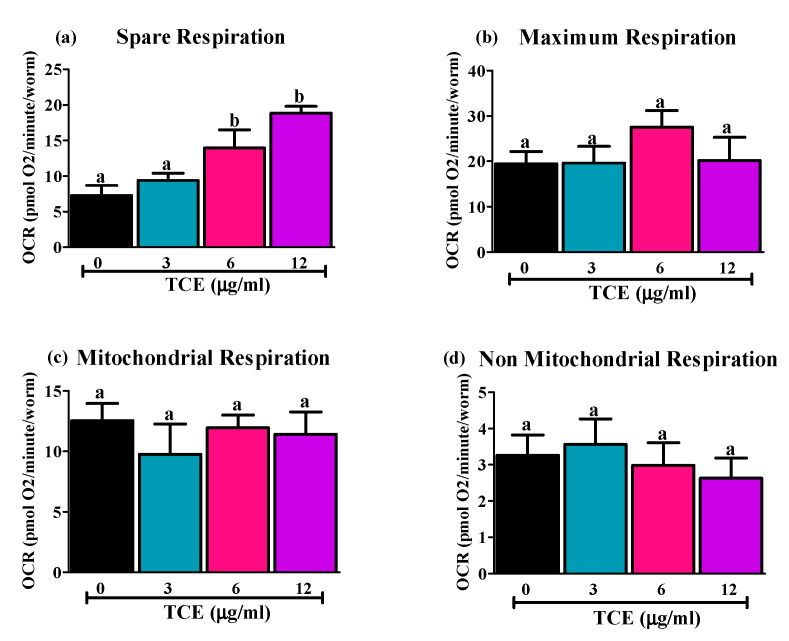
Effects of tart cherry extracts (TCE) on respiration capacity in *C. elegans*. WT worms were treated with 0, 3, 6, and 12 μg/mL TCE. Sea-horse analysis was used to measure how they utilize oxygen. (**a**) Spare respiration was significantly higher (different letters (a, b) are significantly different, *p* < 0.05, one-way ANOVA, 20 worms/well in 4 replicates, *n* = 3 independent experiments) in 6 and 12 μg/mL TCE treated worms compared to 0 and 3 μg/mL treated worms. No difference was observed in (**b**) maximum respiration, (**c**) non-mitochondrial, or (**d**) mitochondrial respiration in TCE treated worms compared to non-treated worms.

As few mitochondrial genes are changed by TCE, we next assessed changes in mitochondrial function/respiration by TCE in *C. elegans*, using the Seahorse Extracellular Flux analyzer, (Figure 3). Only spare respiration, which reflects “the ability of an organism to respond to increasing energy demands” [50] was significantly higher in 6 and 12 μg/mL treated worms compared to non-treated (0 μg/mL) and 3 μg/mL TCE treated worms (Figure 3a). There were no differences observed in maximum (Figure 3b), mitochondrial (Figure 3c), or non-mitochondrial (Figure 3d) respiration. 

### 3.4. TCE Did Not Extend Mean Lifespan in Daf-16 and Aak-2 Mutant Worms

**Figure 4:**
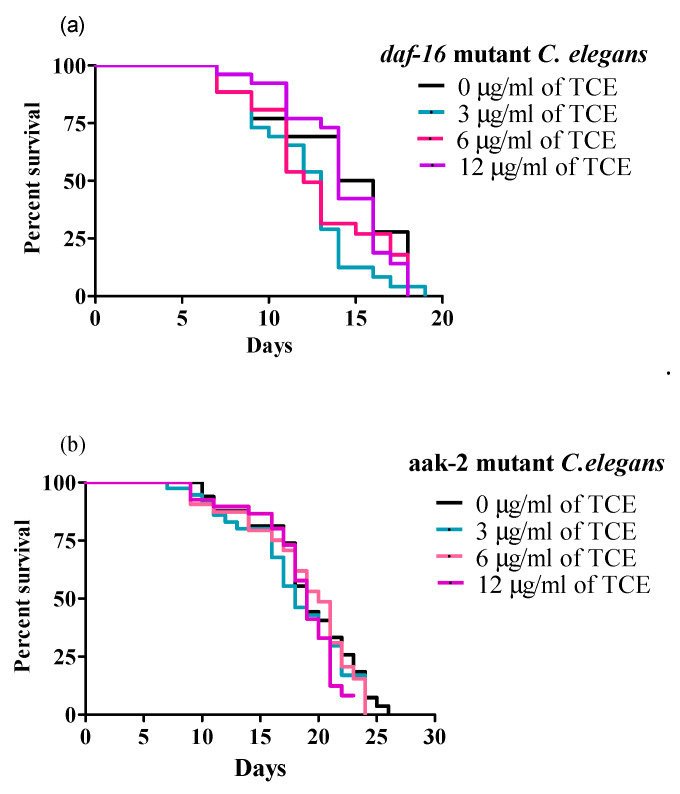
Tart cherry extract (TCE) did not extend lifespan in *daf-16* and *aak-2* mutant *C. elegans*. (**a**) Treatments with 3 µg/mL (blue), 6 µg/mL (pink), and 12 µg/mL (purple). TCE had mean lifespans (MLS) of 12.65 ± 0.43, 13.96 ± 0.51, and 14.29 ± 0.74, respectively in *daf-16* mutant *C. elegans* compared to control or no treatment (0 µg/mL, MLS 13.57 ± 0.51, black) grown at 20 °C. (**b**) Treatments with 3 µg/mL (blue, MLS: 14.83 ± 0.15), 6 µg/mL (pink, MLS: 14.41 ± 0.18), and 12 µg/mL (purple, MLS: 15.64 ± 0.83) TCE did not increase MLS in *aak-2* mutant *C. elegans*, compared to control or no treatment (0 µg/mL, MLS: 15.68 ± 0.20, black) grown at 20 °C. Each lifespan experiment was repeated at least three independent times with similar results. Statistical significance considered at *p* < 0.05 using the log-rank (Mantel–Cox) test.

Because *daf-16* mRNA expression was significantly increased by TCE, we tested whether *daf-16* mutant worms were responsive to the same concentration of TCE used for WT worms to further validate the specific pathways involved in TCE-mediated lifespan extension. None of the TCE concentrations used (3, 6, and 12 μg/mL) significantly changed the MLS of *daf-16* mutant worms (Figure 4a and Supplementary Table S2), which support our initial finding that lifespan extension by TCE is dependent on IIS/DAF-16 pathway. 

Moreover, since TCE significantly increased mRNA expression of *aak-2* in WT worms, and published research showed that both DAF-16 and AAK-2 are involved in regulating longevity [51], we next tested involvement of AAK-2 signaling in lifespan. Interestingly, TCE (3, 6, and 12 μg/mL) did not increase the MLS of *aak-2* mutant worms (Figure 4b and Supplementary Table S3), indicating importance of this pathway in TCE mediated longevity effects.

## 4. Discussion

Studies have observed that bioactive compounds from various botanicals offer a variety of health benefits against obesity, cancer, inflammation, and age-related functional decline [23,35,52,53]. Here, we showed that Montmorency Tart Cherry is a potentially effective nutraceutical for promoting healthy aging in *C. elegans*. We showed that TCE consumption substantially extends lifespan mainly through IIS and DAF-16 signaling. We further confirmed these findings in mutant worms lacking functional *daf-16* and *aak-2* genes. TCE treatment did not extend the lifespan of *daf-16* and *aak-2* mutant worms.

### 4.1. TCE Increases Lifespan through IIS and DAF-16 Pathway

In this study we used 3, 6, and 12 µg/mL of TCE to investigate its longevity effects in *C. elegans*. Previous studies from our lab have shown that TC increased cell survival and antioxidant activity in vitro using adipocytes [35]. Moreover, TC reduced toxic levels of ROS in both adipocytes and Zucker fatty rats [35]. To our knowledge, there are no studies that have documented lifespan effects of TCE in mammalian and only one study has showed TCE effects in model organisms [54]. Some studies have shown that TC improves memory, autophagy, and hippocampal inflammation in aged rats [52] and protects against age-related bone loss in mice [55]. Studies in *C. elegans* with blueberries (50, 100, and 200 mg/mL of extract) demonstrated increased MLS by 22.2%, 36.5%, and 44.4% [27] and 5 mg/mL of blueberry extracts extended MLS of *Drosophila* by 10% [17]. Similarly, cranberry extracts (2 mg/mL) increased MLS of *C. elegans* by 32.5% [13] and 20 mg/mL cranberry extracts extended MLS of *Drosophila* by 10% [8]; purple wheat anthocyanins (100 μg/mL) increased MLS of *C. elegans* by 10.5% [56]. In our study, we found that feeding *C. elegans* lower concentrations of TCE (3, 6, and 12 μg/mL) resulted in significant extension of MLS by 12.09% (*p* > 0.05), 26.44% (*p* < 0.05), and 25.69% (*p* < 0.05), respectively. These results are comparable to findings from the studies outlined above using other plant or fruit extracts [13,27,56]. However, previous studies in *C. elegans* were done on agar plates, which require typically very high doses to get enough drug into the worms, as the drug can diffuse into the agar, and drug delivery is not efficient. Our cultures are in liquid environment due to microfluidics (NemaLife), which is a novel approach to culture worms with several advantages: worms can crawl in an optimized micropillar arena in liquid (unlike in agar plates) that improves drug delivery; during maintenance, sieve channels dispersed progeny, thus avoiding loss of adults; they can feed the adult-only population [41]. Consistent with the advantages mentioned above, observation of lifespan-extension with low doses of TCE suggests that the microfluidic environments are efficient at drug delivery compared to agar plates.

IIS pathway modulates aging and longevity and highly conserved in invertebrates and mammals. Insulin like peptide (ILPs), an insulin/IGF-1 receptor (DAF-2), serine/threonine kinase (pyruvate dehydrogenase kinase isozyme 1 (PDK-1), AKT-1/2, AGE-1)/PI3K and FOXO transcription factor (DAF-16) are major components of the IIS transduction cascades [49]. Inactivation of DAF-16 transcription factor occurs after binding of ILPs to DAF-2, inhibiting the nuclear translocation of DAF-16 [57], which negatively regulates longevity. 

In addition to IIS signaling, many signaling pathways such as TOR, AMPK, Ca^2+^/calmodulin-dependent protein kinase II (CaMKII), and c-Jun N-terminal kinase (JNK) pathways are involved in aging [49,58]. Inhibition of TOR signaling increases lifespan of *C. elegans* and *Drosophila* via DAF-16 [8,59], CaMKII targets DAF-16 and modulates lifespan when worms are exposed to heat, hunger, or some other form of stressors [58], and JNK-1 signaling also regulates the activity of DAF-16 to promote lifespan post transcriptionally [60]. 

Very limited studies have tested effects of bioactive compounds in worms. Supplementation with wild blueberry juice (50, 100, and 200 mg/mL) increased *C. elegans* lifespan through CaMKII pathway that mediates osmotic stress resistance [28]. Other studies using fresh blueberry (200 μg/mL) also showed increased lifespan and stress tolerance through DAF-16 [27], cranberry extract (2 mg/mL) [25] and anthocyanin-rich purple wheat extract (100 μg/mL) [56] increased lifespan in *C. elegans* via the IIS/DAF-16 signaling pathway [13,25,56]. Further studies showed that oleanolic acid increased lifespan and stress tolerance through DAF-16 [61] while resveratrol, found in grape berry skin (100 μM), increased lifespan in *C. elegans* through sirtuin 2.1 (SIR 2.1), mammalian NAD-dependent protein deacetylase homology [62]. Moreover, blackberry extract increased antioxidant capacity in worms and provided protection against ROS generation [63]. In addition, a recently published study revealed that TCE acts as a calorie restriction mimetic and increased lifespan in *C. elegans* [54].

Our findings showed that TCE regulates many components in IIS pathway to influence lifespan. TCE did not increase lifespan in daf-16 mutant *C. elegans*, indicating that TCE may regulate lifespan through DAF-16. Further analysis of genes in WT worms also confirmed that TCE regulates lifespan mainly via DAF-16/IIS pathway, evident by significantly reduced expression of *daf-2* and increased expression of *daf-16* genes. Since both DAF-2 and DAF-16 in IIS pathway are highly conserved, our findings reveal the significant potential of TCE to promote healthy aging in humans. Although we did not see any changes of *akt-1* and *lin-14*, two inhibitors of DAF-16, at mRNA levels, there are many other upstream pathways and downstream targets that we did not test. Thus, it is possible that TCE regulates upstream molecular targets of *akt* and *lin-14*, which merit further investigation, including those that control the expression of these genes such as *pdk-1* and *lin-4*. SKN-1 is another transcription factor, which acts downstream of IIS pathway and showed reduced expression with reduced IIS signaling [64]. We found that *skn-1* gene expression was also upregulated by TCE treatments in WT worms. Our results are consistent with a recently published study using *Hibiscus sabdariffa* L. extracts which increased SKN-1 activity and prolonged lifespan in *C. elegans* [65]. Studies have shown that cherries including sour cherry possess antimicrobial activity [66]. We performed all our gene expression data in liquid CMEM media without bacteria supplementation and still observed increased expression of lifespan-related genes. However, in our current study, we did not measure anti-microbial activity of TCE on the OP50 bacteria which might account for part of calorie restriction-like effects on *C. elegans* to increase lifespan. Therefore, these findings must be validated in future in lifespan assays.

### 4.2. Involvement of AMPK/AAK-2 in Lifespan Determination

Interestingly, TCE did not increase the MLS of *aak-2* mutant worms, indicating importance of this pathway in TCE-mediated longevity effects. AMP-activated protein kinase (AMPK) is a fuel-sensing enzyme that negatively regulates fatty acid synthesis and positively regulates energy production pathways such as glycolysis and fatty acid β-oxidation [57]. Activation of AAK-2, homolog of AMPK in *C. elegans* physiologically (dietary restriction) or pharmacologically (resveratrol or metformin), increased lifespan of *C. elegans* [67]. *C. elegans* has both *aak-1* and *aak-2*, two different genes. The AMPKα1 subunit in *C. elegans*, AAK-2, is activated by AMP and involved in longevity [51]. By contrast, AMPKα2 subunit, AAK-2, in *C. elegans* is not involved in longevity effects suggesting that AAK-2 is the subunit with kinase activity [67]. Because of that, here, we evaluated the effects of TCE in *aak-2* worm lifespan and *aak-2* gene expression in WT worms. TCE did not change the MLS of *aak-2* worms treated with TCE. Previous studies reported that *aak-2* (ok524) mutants have a shorter lifespan (12%) than WT worms [51]. In contrast to these findings, however, *aak-2* (rb754) mutant worms we used for our lifespan assays did not show shorter average lifespan than WT worms, and *aak-2* (rb754) had 17.8% increased lifespan than WT worms. The above reported study has been conducted on agar plates, while we used microfluidic devices for the lifespan assay and fed worms everyday with liquid OP50 mixed with TCE. Changes of the environment, use of microfluidic devices, and differences in the *aak-2* strains used might be a potential cause for these discrepancies. Further genetic analysis in WT worms showed increased expression of *aak-2* gene with TCE treatments. Possibly, activation of *aak-2* increased fatty acid oxidation in WT worms, which may change total fatty acid composition in the worms and indirectly influence the lifespan of WT worms. However, these changes were not taking place in mutant *aak2*, which may affect their responses to TCE and changes in fatty acid composition or lipid metabolism. These findings suggest that TCE acts partly through AAK-2 pathway and requires DAF-16 and IIS pathways to increase lifespan in *C. elegans*. Sirtuins-1 (*Sirt-1*), an ortholog of mammalian yeast deacetylase *Sir-2* linked to IIS signaling pathway [12], may also be involved in lifespan regulation and merits further investigation.

### 4.3. Role of Mitochondria in Lifespan Determination

Mitochondria plays an important role in overall cellular and organism health. Mitochondria are involved in cellular metabolism, apoptosis, and production of ROS [60]. It is thought that impaired mitochondria produce more ROS which causes cellular damage and leads to aging [68,69]. SOD is an enzyme that is involved in detoxification [16,70] and is found in cytoplasm, nucleus, and mitochondria [71]. Experiments in yeast and mice showed that knocking out *sod-1* and *sod-2* decreased lifespan [72,73]. In contrast, another study reported that SOD has no effect on lifespan in *C. elegans* [16]. In our current study, *sod-2* gene levels were increased with TCE, with no changes in *sod-3* gene expression. Another study using purple pitanga fruit showed increased *sod-3* protein expression in *C. elegans* [19]. We chose *sod-3* over *sod-1* as they share some genetic similarities and have a certain level of amino acid homology, and *sod-2* does not share substantial amino acid homology with either *sod-1* or *sod-3* [74]. Possibly, increased *sod-2* gene expression in our study decreased mitochondrial ROS activity and extended lifespan. However, these findings remain to be confirmed using *sod-2* mutant worms. Furthermore, to validate effects of TCE on ROS production, we plan in future to conduct additional functional assays to measure ROS, GSH/SG, and MDA levels. 

Ideal mitochondrial function is critical to have healthy cellular activity, and mitochondrial respiration is a strong indicator of good mitochondrial function [75]. An age-related decline in oxidative phosphorylation can relate to reduced mitochondrial and nuclear expressed peptides functioning in the electron transport chain (ETC) [76]. Mitochondrial health can be measured by the rate of mitochondrial respiration in ETC [77,78]. Moreover, alterations in the oxygen consumption rate are an indicator of mitochondrial dysfunction [79]. Moreover, decreased mitochondrial function reduces sufficient energy production, which plays key role during aging [80]. In order to quantify the function of mitochondria, we assessed mitochondrial OCR by TCE treatments in WT *C. elegans*. Our data showed that TCE treated adult worms have significantly higher spare respiration capacity with 6 and 12 μg/mL concentrations. Spare respiratory capacity is the amount of additional ATP produced by oxidative phosphorylation as a result of sudden increase in energy demand [76]. Based on our findings, it is possible that increased expression of antioxidant genes, led to increased mitochondrial spare respiration by reducing ROS production and oxidative stress in part through *sod-2*. 

Another indicator of mitochondrial function is uncoupling proteins (UCPs). *C. elegans* expresses *ucp-4*. UCPs are highly conserved in many species, such as invertebrates, plants, and mammals. UCP-1 in mammals is induced by cold temperature and is a major player in thermogenesis and energy expenditure [81]. Mammals express several other UCPs, which are involved in different functions other than thermogenesis. It is reported that non-thermogenic UCPs protect cells against oxidative stress [82]. In support of these ideas, knocking down *ucp-2* gene in mice generated more ROS than WT mice [83], and knocking down *ucp-3* gene led to more ROS production in skeletal muscle [84]. UCP-like protein in *C. elegans* is thoroughly related to *ucp-4* in mammals, and the two proteins share 46% sequence identity [85]. Here, we found that expression of *ucp-4* was significantly higher in worms treated with 6 and 12 μg/mL TCE. Several studies documented that *ucp-4* mutants may be involved in increased neuronal defects during aging [68,86], but none of the studies have reported lifespan extension effects of *ucp-4* by reducing mitochondrial ROS production. Our findings suggest that reduced ROS production and increased mitochondrial function by TCE may be governed partially by increased *ucp-4* expression.

## 5. Conclusions

**Figure 5:**
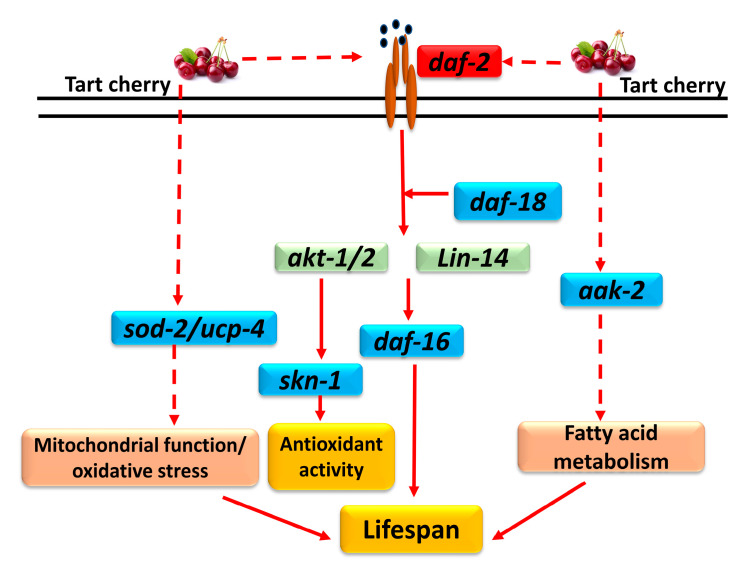
Major molecular targets for TCE in *C. elegans*. TCE downregulated *daf-2* gene and up-regulated *daf-16*, *daf-18*, *skn-1*, *sod-2*, *ucp-4*, and *aak-2* while no changes were observed in *akt-1* or *lin-14* genes. These molecules may be indirectly involved in regulating mitochondrial function, oxidative stress, ant-oxidant activity, and fatty acid metabolism, and this may subsequently increase lifespan of *C. elegans*. Downregulated genes are shown in red; up-regulated genes are shown in blue, and those genes that were not changed by TCE are shown in green. Dotted arrows show potential indirect targets of TCE.

In conclusion, we have shown that TCE increases lifespan of *C. elegans*, in a DAF-16-dependent manner. This may be mainly due to genetic regulation in IIS and DAF-16 pathway, partial involvement of AAK-2 signaling, increased antioxidant activity, and spare respiratory activity, allowing cells to function better under stressful conditions. Major underlying molecular targets are summarized in Figure 5, some of which require additional validations. Further studies are warranted to understand mechanisms linking oxidative stress, respiration, and longevity and their regulation by dietary antioxidants such as tart cherry anthocyanins. 

## Supplementary Materials

The following are available online at https://www.mdpi.com/2072-6643/12/5/1482/s1. Table S1, the lifespan in wild type *C. elegans* with different concentrations of tart cherry at 20 °C; Table S2, the lifespan in daf-16 mutant *C. elegans* with different concentrations of tart cherry at 20 °C; Table S3, the lifespan in aak-2 mutant *C. elegans* with different concentrations of tart cherry at 20 °C.
