# Supplementary material for: Tart Cherry Increases Lifespan in Caenorhabditis elegans by Altering Metabolic Signaling Pathways
Source: Nutrients. 2020 May 20;12(5):1482. doi: 10.3390/nu12051482 (PMC7285199; doi:10.3390/nu12051482)
Supplement: Supplementary file 1 [file nutrients-12-01482-s001.pdf]

SUPPLEMENTARY TABLES

Table S1: The lifespan in wild type *C. elegans* with different concentrations of tart cherry at 20⁰C.

Treatment	Mean ± SE	Median lifespan	Maximum lifespan	Total number of worms
**0 μg/ml**	13.31 ± 0.61[a]	14	19	120
**3 μg/ml**	14.92 ± 0.58[a]	15	22	120
**6 μg/ml**	16.83 ± 0.77[b]	17	22	120
**12 μg/ml**	16.73 ± 0.67[b]	17	21	120

Lifespan assay was performed in micro-fluidic devices at 20⁰C, with (3, 6, or 12 μg/ml) or without (0 μg/ml) tart cherry extract. Each device contained 30 chambers and only one worm per chamber. Chambers were washed everyday with nematode growth medium to avoid progenies. Each lifespan experiment was performed 3 independent times with similar results. Values with different letters are significantly different ($p<0.05$) log rank (Mantel-Cox) test.

Table S2. The lifespan in *daf-16* mutant *C. elegans* with different concentrations of tart cherry at 20^{0}C.

Treatment	Mean ± SE	Median lifespan	Maximum lifespan	Total number of worms
**0 μg/ml**	13.57 ± 0.51	14	18	120
**3 μg/ml**	12.65 ± 0.43	13	19	103
**6 μg/ml**	13.96 ± 0.51	12	19	103
**12 μg/ml**	14.29 ± 0.74	14	19	107

Lifespan assay was performed in micro-fluidic devices at 20^{0}C, with (3, 6, or 12 μg/ml) or without (0 μg/ml) tart cherry extract. TC extract did not change the mean lifespan of *daf-16 C. elegans* significantly. Each device contained 30 chambers with only one worm per chamber. Chambers were washed everyday with nematode growth medium to avoid progenies. Each lifespan experiment was performed 3 independent times with similar results. Values with different letters are significantly different ($p<0.05$) log rank (Mantel-Cox) test.

Table S3. The lifespan in *aak-2* mutant *C. elegans* with different concentrations of tart cherry at 20^{0}C.

Treatment	Mean ± SE	Median Lifespan	Maximum lifespan	Total number of worms
**0 µg/ml**	15.68 ± 0.20	19	26	90
**3 µg/ml**	14.83 ± 0.15	18	24	100
**6 µg/ml**	14.41± 0.18	20	24	103
**12 µg/ml**	15.64 ± 0.83	19	23	104

Lifespan assay was performed in micro-fluidic devices at 20^{0}C, with (3, 6, or 12 µg/ml) or without (0 µg/ml) tart cherry extract. TC extract did not change the mean lifespan of *aak-2 C. elegans* significantly. Each device contained 30 chambers and only one worm per chamber. Chambers were washed everyday with nematode growth medium to avoid

progenies. Each lifespan experiment was performed 3 independent times with similar results. Values with different letters are significantly different ($p<0.05$) log rank (Mantel-Cox) test.
